# Updated perspective of EPAS1 and the role in pulmonary hypertension

**DOI:** 10.3389/fcell.2023.1125723

**Published:** 2023-02-27

**Authors:** Na Wang, Jing Hua, Yuhua Fu, Jun An, Xiangyu Chen, Chuancui Wang, Yanghong Zheng, Feilong Wang, Yingqun Ji, Qiang Li

**Affiliations:** ^1^ Department of Pulmonary and Critical Care Medicine, Shanghai East Hospital Affiliated by Tongji University, Shanghai, China; ^2^ Department of Pulmonary and Critical Care Medicine, Central Hospital of Jiading District, Shanghai, China; ^3^ Department of Pulmonary and Critical Care Medicine, First Affiliated Hospital of Dalian Medical University, Dalian, China; ^4^ Department of Pulmonary and Critical Care Medicine, Jinshan Branch of Shanghai Sixth People’s Hospital, Shanghai, China

**Keywords:** EPAS1, pulmonary hypertension, hypoxia, vascular remodeling, transcription factor

## Abstract

Pulmonary hypertension (PH) is a group of syndromes characterized by irreversible vascular remodeling and persistent elevation of pulmonary vascular resistance and pressure, leading to ultimately right heart failure and even death. Current therapeutic strategies mainly focus on symptoms alleviation by stimulating pulmonary vessel dilation. Unfortunately, the mechanism and interventional management of vascular remodeling are still yet unrevealed. Hypoxia plays a central role in the pathogenesis of PH and numerous studies have shown the relationship between PH and hypoxia-inducible factors family. EPAS1, known as hypoxia-inducible factor-2 alpha (HIF-2α), functions as a transcription factor participating in various cellular pathways. However, the detailed mechanism of EPAS1 has not been fully and systematically described. This article exhibited a comprehensive summary of EPAS1 including the molecular structure, biological function and regulatory network in PH and other relevant cardiovascular diseases, and furthermore, provided theoretical reference for the potential novel target for future PH intervention.

## 1 Introduction

Pulmonary hypertension (PH) is a group of diseases caused by various etiologies, including genetic mutations and environmental and toxin exposures, that results in pulmonary vascular remodeling and the subsequent elevation of vascular resistance and pressure and right ventricular overload, ultimately leading to right heart decompensation and death (Zheng et al., 2020). Clinically, PH is categorized into five groups: pulmonary arterial hypertension (PAH), PH associated with left heart disease, PH associated with lung disease and/or hypoxia, PH associated with pulmonary artery obstructions, and PH with unclear or multifactorial mechanisms (Hoeper et al., 2016; Humbert et al., 2022). The diagnostic criteria for PH were updated as a mean pulmonary artery pressure (mPAP) > 20 mmHg at rest during right heart catheterization according to the 2022 European Society of Cardiology/European Respiratory Society PH guidelines (Humbert et al., 2022). The primary pathophysiology of pulmonary vascular remodeling involves the intimal endothelial cell (ECs) proliferation and apoptotic resistance, medial smooth muscle cell (SMCs) hypertrophy and proliferation, adventitial fibroblast proliferation and activation with excessive extracellular matrix (ECM) deposition, and interstitial or perivascular inflammatory infiltration (Tuder et al., 2013a; Tuder et al., 2013b; Galiè et al., 2019). Although multiple pharmacological therapies have been developed over the past few decades that mainly target vasomotor tones such as endothelin (ET), prostaglandin I_2_, and nitric oxide (NO)/cyclic guanosine monophosphate pathways (Zheng et al., 2020), PH patients have a relatively low survival rate and high mortality, about 57% (Benza et al., 2012; Tuder et al., 2013a; Tuder et al., 2013b; He et al., 2020), due to the complexity of etiologies and limited interventions for irreversible pulmonary vascular remodeling process.

**FIGURE 1:**
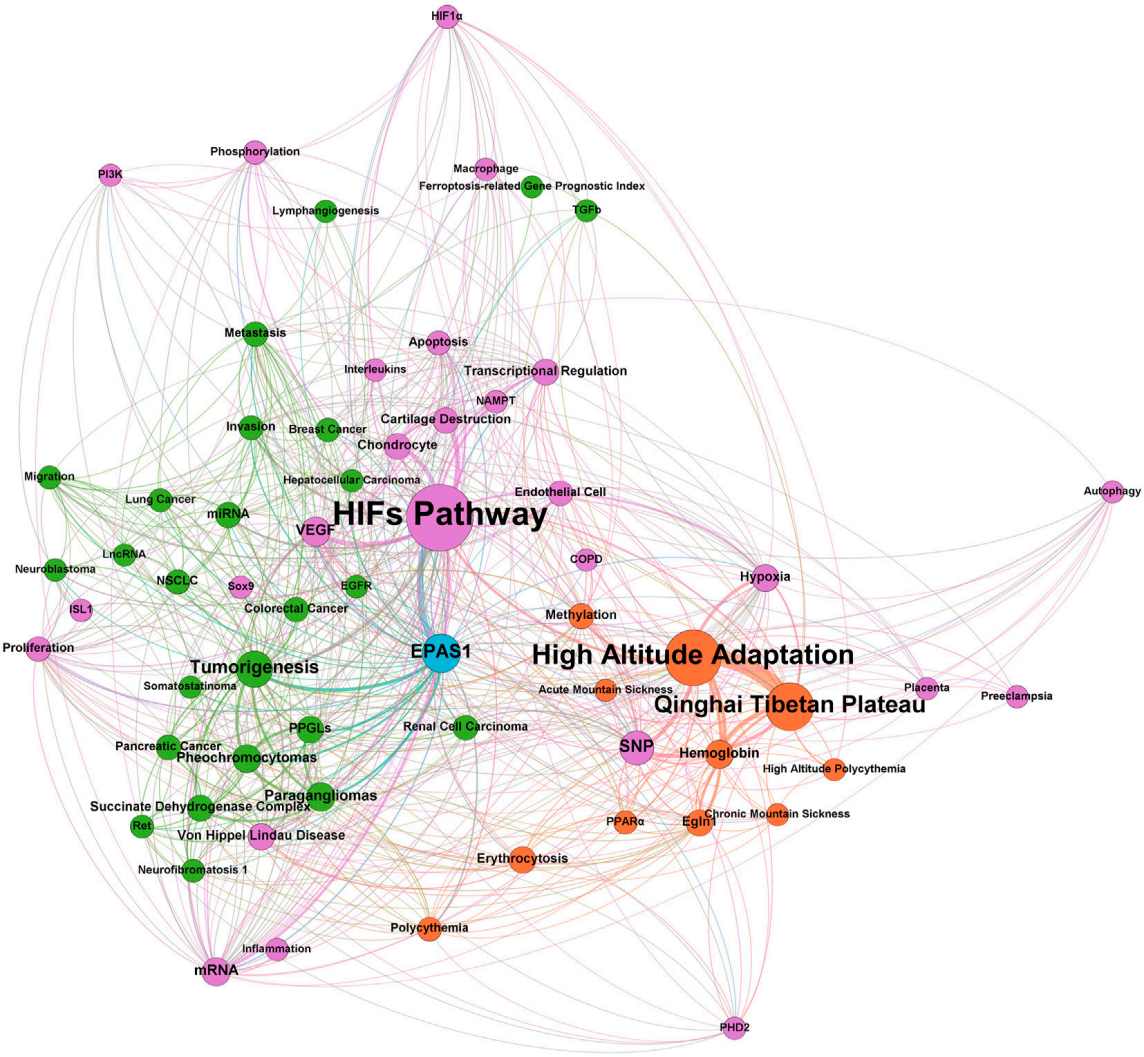
Bibliometrics analysis of EPAS1 in the past 20 years.

Endothelial PAS domain-containing protein 1 (EPAS1), also known as hypoxia-inducible factor 2 alpha (HIF-2α), is a protein encoded by the *EPAS1* gene on chromosome 2 of humans and almost exclusively expressed by ECs (Young et al., 2019). According to the bibliometric analysis (Figure 1), studies in the past 20 years revealed the transcriptional regulatory role of HIF-2α in different cellular pathways including hypoxic metabolism, inflammation, apoptosis, and angiogenesis (Luo et al., 2011), as well as various correlated diseases, including pheochromocytomas, Von-Hipple-Lindau disease, and other malignancies. The most specific pathological condition relevant to HIF-2α is high-altitude environmental adaptation, such as acute or chronic mountain sickness and subsequent secondary polycythemia with erythrocytosis.

Furthermore, consecutive publications have illustrated the pathological role of *EPAS1* in the development of PH in the past decade, but its mechanism of action has not been thoroughly explored. Therefore, this review aims to provide a comprehensive summary of *EPAS1* in the pathogenesis and potential therapeutic interventions of PH.

## 2 Structure and regulation

As first identified by Semenza in Hep3 cells in 1992 (Semenza and Wang, 1992), the hypoxia-inducible factor (HIF) family is a group of transcription factors involved in the intracellular response sensing oxygen concentration, which is strongly associated with organism growth and development and disease pathogenesis.

**FIGURE 2:**
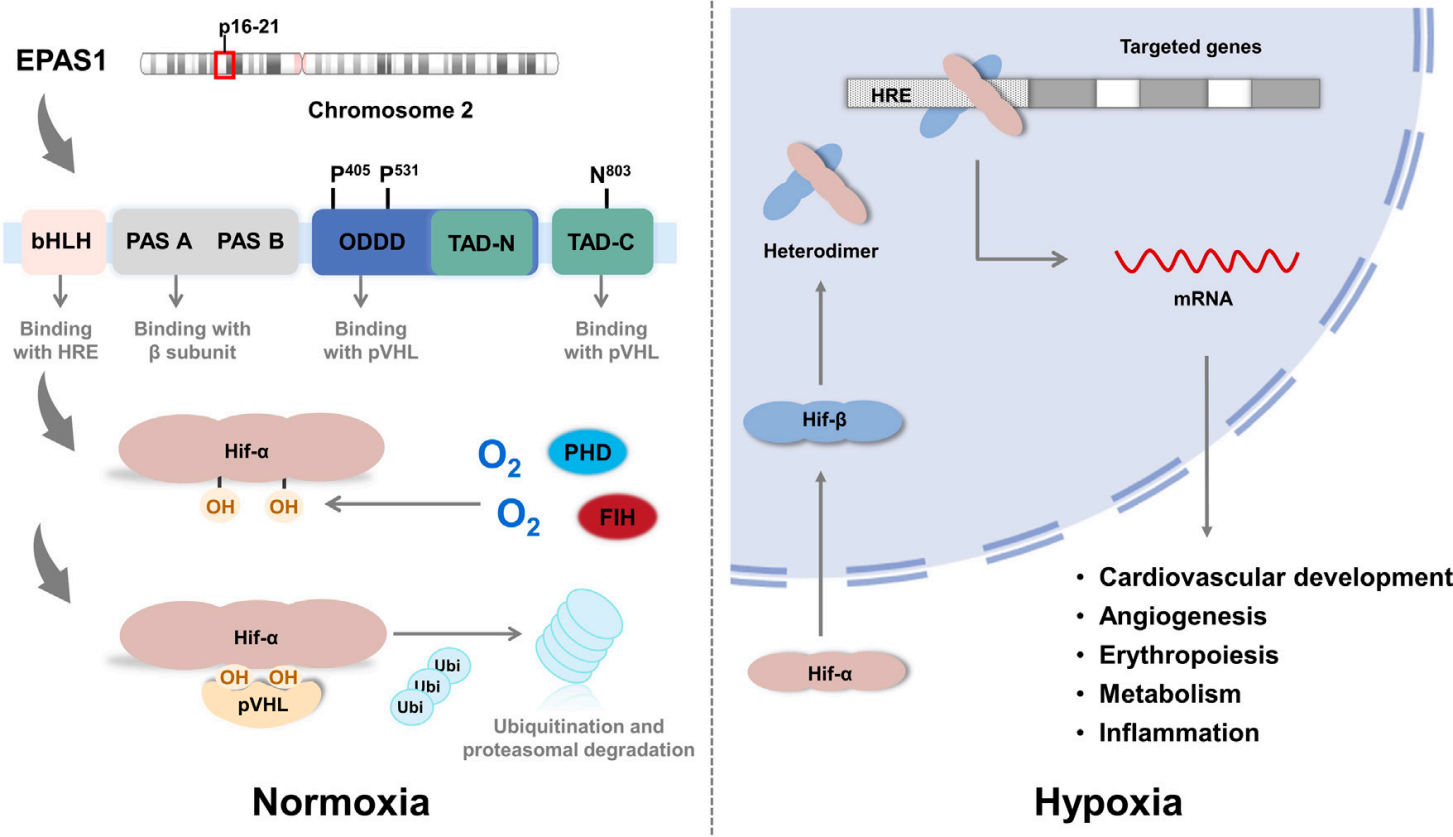
The structure of EPAS1.

Structurally, HIF is a heterodimer composed of an oxygen-sensitive alpha subunit and constitutively expressed beta subunit (Figure 2). Both alpha and beta subunits belong to the PER-ARNT-SIM (PAS) subfamily of basic helix-loop-helix (bHLH) transcription factors. *EPAS1* is located on chromosome 2 (specifically p16–21 regions), shares 48% homology of the primary amino acid sequence with HIF-1α(Tian et al., 1997), and contains the following domains (Yang et al., 2005; Zhao et al., 2015): 1) a bHLH domain at the *N*-terminal for DNA binding to hypoxia-responsive elements (HREs) in the promoters or enhancers of target genes (Lim et al., 2013); 2) a PAS domain that facilitates heterodimer formation with beta subunit; 3) the oxygen-dependent degradation domain (ODDD) is responsible for the hydroxylation of proline residues (Pro-405 and Pro-531) and degradation under normoxic conditions; and 4) two transcriptional activation domains (TADs) recruit and interact with transcriptional coregulatory proteins such as CREB-binding protein/E1A binding protein p300 (CBP/p300) to maintain transcriptional activity. *N*-terminal TAD (N-TAD) lies within the sequence of ODDD, which confers specific target genes to HIF-2α, whereas C-terminal TAD (C-TAD) is responsible for the common target genes of HIF-1α (Daly et al., 2021).

Under sufficient oxygen availability, HIF alpha subunits are hydroxylated at conserved proline residues by HIF prolyl-hydroxylases (PHD), allowing for the recognition and ubiquitination by von Hippel-Lindau (VHL) E3 ubiquitin ligase, leading to degradation by the proteasome (Daly et al., 2021). HIF prolyl-hydroxylases are inhibited under hypoxic conditions because of the limited oxygen utilization as a cosubstrate, stabilizing the alpha subunit from ubiquitination and allowing it to translocate into the nucleus and form a transcriptionally active heterodimer with the beta subunit. This complex then binds to the HRE regions of the promoters of its downstream genes (Wang et al., 1995), which are involved in various physiological processes, including cardiovascular development, metabolism, inflammation, angiogenesis, and erythropoiesis, that are believed to be associated with PH development (Kim and Yang, 2015; Yoo et al., 2015). Notably, in the study of Petra Miikkulainen (Miikkulainen et al., 2019), a strong positive correlation between HIF-prolyl hydroxylase 3 (PHD3) and HIF-2α mRNA expression in renal clear cell carcinomas (RCCs) is observed, in contrast to the expected accumulation of HIF-2α after PHD3 knockdown in non-RCC cells, which also indicates the aggressiveness and poor prognosis of RCC. In addition to PHD-pVHL pathway, factor inhibiting HIF (FIH) participates the hydroxylation of asparagine-803 in the CTAD domain of HIF-2α and inhibits interactions with transcriptional coactivators such as CBP/p300 (Lando et al., 2002; Greer et al., 2012). Another pathway participating in the degradation of HIF-2α protein is PTEN/PI3K/AKT (Joshi et al., 2014), which phosphorylates E3 ligase of VHL and inactivates the ubiquitination in tumor associated macrophages (TAMs). In terms of transcription, studies revealed that the expression of HIF-2α is positively regulated by IGF induced PI3K-mTORC2 signaling (Mohlin et al., 2015) and peroxisome proliferator-activated receptor gamma coactivator (PGC)/Estrogen-related receptor (ERR) complex families (Hamidian et al., 2015) in neuroblastoma cells; IL-4 signal in macrophages (Takeda et al., 2010); deubiquitylase Cezanne (also known as OTUD7B) by stabilizing transcription factor E2F1 in various cell lines (Moniz et al., 2015); and suppressed by histone deacetylases (HDACs) in soft tissue sarcoma (Nakazawa et al., 2016). An anti-inflammatory cytokine 15-Deoxy-Delta-12,14-prostaglandin J2 (15d-PGJ2), identified by Michael Zimmer (Zimmer et al., 2010) and colleagues, inhibits HIF-2α translation by promoting the binding of iron regulatory protein-1 (IRP1) to iron responsive element (IRE) of HIF-2α message and exerts the anti-inflammatory and putative antineoplastic effects. Other pathways including post-translational modifications and miRNAs interference (Gradin et al., 2002; To et al., 2006; Dioum et al., 2009; Van Hagen et al., 2010; Chen et al., 2012; Poitz et al., 2013; Mathew et al., 2014; Zhang et al., 2014; Sohn et al., 2015; Pangou et al., 2016) are summarized in Table 1, these data disclose the complex regulatory networks of HIF-2α pending further completion.

**TABLE 1:** Regulations of HIF-2α.

Regulation	Pathways	Effect	References
Transcription	PHD3	Promotes transcription	Miikkulainen et al. (2019)
	IGF-PI3K-mTORC2	Promotes transcription	Mohlin et al. (2015)
	PGC/ERR complex	Promotes transcription	Hamidian et al. (2015)
	IL-4	Promotes transcription	Takeda et al. (2010)
	Cezanne-E2F1	Promotes transcription	Moniz et al. (2015)
	HDACs	Suppresses transcription	Nakazawa et al. (2016)
MicroRNA	miR-30c-2-3p	Suppresses transcription	Mathew et al. (2014)
	miR-30a-3p	Suppresses transcription	Poitz et al. (2013)
	miR-17	Suppresses transcription	Zhang et al. (2014)
	miR-20a	Suppresses transcription	Sohn et al. (2015)
Translation	15d-PGJ2	Inhibits translation	Zimmer et al. (2010)
Post-translation	PHD-VHL	Promotes degradation	Daly et al. (2021)
	FIH	Promotes degradation	Lando et al. (2002); Greer et al. (2012)
	PTEN/PI3K/AKT	Inhibits degradation	Joshi et al. (2014)
	Phosphorylation	Inhibits degradation	Gradin et al., 2002; To et al., 2006; Pangou et al., 2016
	SUMOylation	Promotes degradation	van Hagen et al., 2010
	Acetylation	Promotes signals	Chen et al., 2012; Dioum et al., 2009

## 3 Biological function

### 3.1 Cardiovascular development and angiogenesis

**FIGURE 3:**
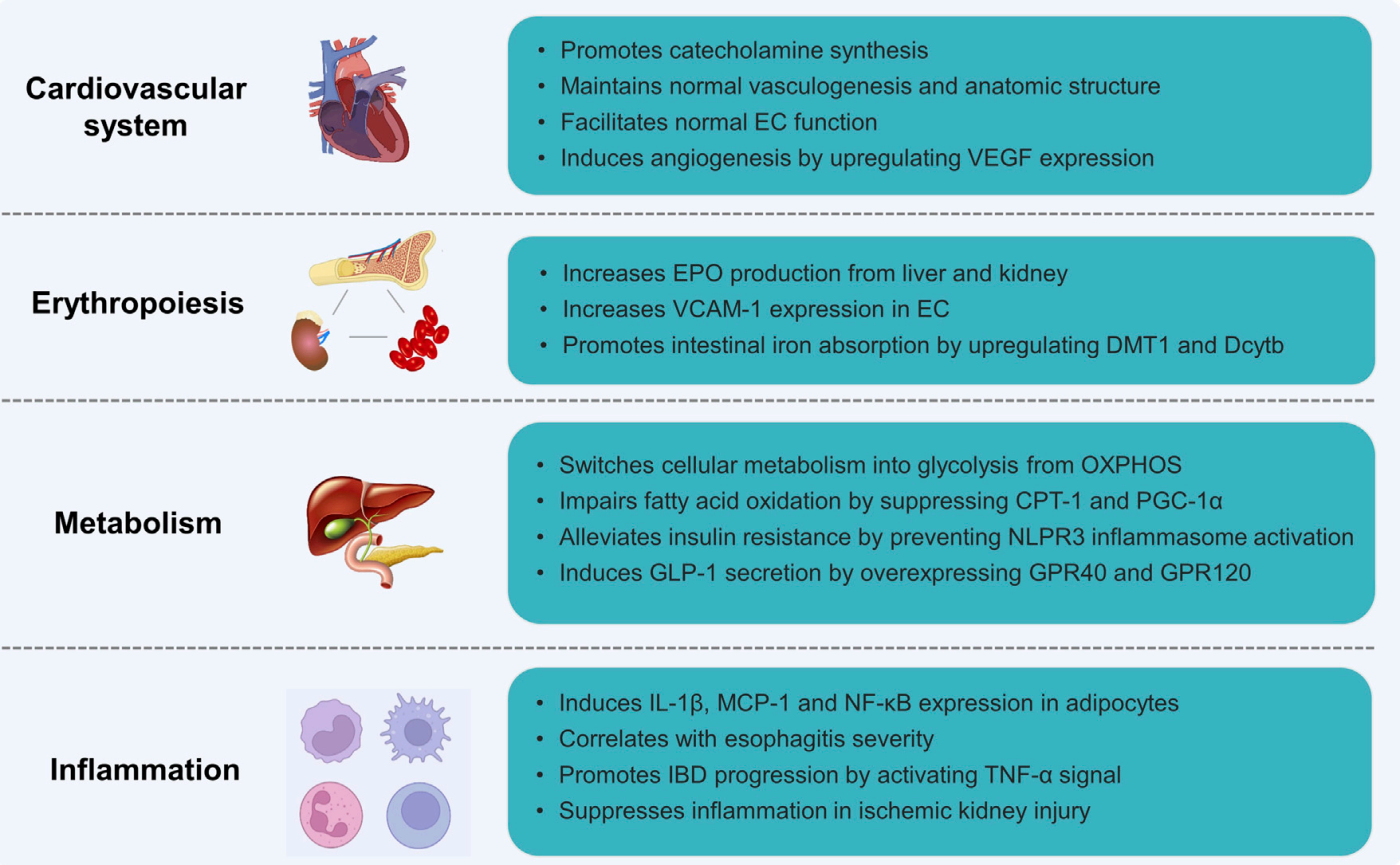
The biological functions of EPAS1.

*EPAS1* was first found by Tian (Tian et al., 1997) in 1997 as a transcription factor exclusively expressed in vascular ECs of the umbilical cord that regulates vascularization and response to hypoxia. The following year, Tian (Tian et al., 1998) further reported that *EPAS1* is essential for the maintenance of cardiac output and circulating catecholamine levels from the endothelium under hypoxic conditions during embryonic development. During early embryonic development, as shown in Figure 3, HIF-2α is maintained at a high level in the chromaffin cells of the Organ of Zuckerkandl, the major source of fetal catecholamines. Homozygous *EPAS1*-deficient mice failed to survive in the mid-gestational stage owing to profound bradycardia and circulatory failure. Additionally, HIF-2α plays an indispensable role in angiogenesis compared to HIF-1α, which mainly activates the glucose metabolic pathways (Wang et al., 2005). Peng (Peng et al., 2000) found that *EPAS1*-deficient embryos failed to form large vessels or seal intact structures in the yolk sac, indicating improper vascular remodeling during vasculogenesis. Vadive (Vadivel et al., 2014) reported arrested growth of pulmonary vessels and alveoli after *EPAS1* expression and HIF-1α was reduced by dominant-negative adenovirus-mediated gene transfer or chetomin. Mice with EC-specific *EPAS1* deletion showed increased vascular permeability with ultrastructural abnormalities despite normal vascular anatomic development, resulting in PH(Skuli et al., 2009). Furthermore, *EPAS1* regulates angiogenesis in various solid malignancies of the gastrointestinal and genitourinary tracts, such as colonic/hepatic/pancreatic cancer and breast/ovarian/prostate/renal carcinomas (Blancher et al., 2000; Palayoor et al., 2003; Zhang and Rigas, 2006; Osada et al., 2007; Bertout et al., 2008; Imamura et al., 2009; Menrad et al., 2010) by promoting ECs germination, migration, and adherens junctions (Majmundar et al., 2010; Kovacic et al., 2012; Park et al., 2013).

Vascular endothelial growth factor (VEGF) is believed to be the primary target of HIF-2α during angiogenesis. Dumpa (Dumpa et al., 2019) found that caffeine therapy for premature apnea may reduce the incidence of bronchopulmonary dysplasia by enhancing *VEGF* and angiopoietin expression through the HIF-2α pathway and restoring pulmonary microvasculature and alveolarization in the adult lung. Weidemann (Weidemann et al., 2010) reported that retinal astrocyte-derived VEGF driven by EPAS1 was a key mediator of vascular proliferation in a model of hypoxia-induced retinopathy. Turner (Turner et al., 2002) also revealed consistency in cellular *VEGF* mRNA and HIF-2α protein levels, indicating the pro-angiogenic role of HIF-2α exertion on the HRE sequences of *VEGF* and *VEGF receptors (VEGFR)* and promotion of transcription under hypoxic conditions.

### 3.2 Erythropoiesis

Etiologies triggering excessive erythropoiesis include acute hemorrhage, adaptation to high altitude, or pathological hypoxic conditions such as congenital cyanotic heart diseases and chronic lung diseases (Semenza, 2022). HIF-2α promotes erythropoiesis by regulating erythropoietin (EPO) transcription (Figure 3), a glycoprotein highly expressed in perisinusoidal cells in the liver during embryonic development and produced by interstitial fibroblasts in the kidney during adulthood (Wang et al., 1995; Haase, 2010). HIF-2α recognizes and binds to the core sequence 5′-TACGTGCT-3′ of the *EPO* HRE sequence, which activates transcription and mediates the cellular response to the hypoxic microenvironment (Hussain et al., 2008). In a study by Gruber M (Gruber et al., 2007), both newborn and adult rats with *EPAS1* knockout presented with anemia or severe pancytopenia with suppressed EPO production, indicating the pro-erythrocytosis effect of HIF-2α by transcriptionally regulating EPO production (Scortegagna et al., 2003; Scortegagna et al., 2005). Toshiharu Y (Yamashita et al., 2008) illustrated, however, that HIF-2α governs erythropoiesis by specifically regulating vascular adhesion molecule-1(*VCAM-1*) expression in ECs to maintain the hematopoietic microenvironment compared to normocytic anemia in mice with *EPAS1* knockdown.

In addition to EPO production, HIF-2α stimulates erythropoiesis by participating in intestinal iron absorption, which is in high demand during erythropoiesis and critical for oxygen-carrying red blood cell maturation under hypoxic conditions. Anderson (Anderson et al., 2011) demonstrated that mice with intestinal disruption of *EPAS1* (*EPAS1* ^ΔIE^) showed decreased serum iron levels and expression of iron absorption genes, such as divalent metal transporter 1 (*DMT1*) and Duodenal cytochrome b (*Dcytb*), in phenylhydrazine-induced erythropoiesis. In contrast, an iron responsive element (IRE) is found in the 5′untranslated region of the *EPAS1* sequence, and the translation of the HIF-2α protein can be repressed by the binding of iron regulatory proteins to the 5′IRE of *EPAS1* during iron deficiency (Percy et al., 2007). Additionally, elevated HIF-2α levels during hypoxia suppress hepcidin expression by inducing hepatic EPO production, leading to enhanced intestinal iron uptake and release from internal stores (Liu et al., 2012). Taken together, these data demonstrate the net regulation among *EPAS1*, erythropoiesis, and iron metabolism.

### 3.3 Metabolism

Metabolism is essential for the maintenance of biological activities, and metabolic reprogramming is a believed hallmark of cellular dysfunction of different etiologies (Faubert et al., 2020). Transformation from mitochondrial oxidative phosphorylation (OXPHOS) to cytoplasmic glycolysis is a major adaptive change in response to insufficient oxygen availability (Shaw, 2006). Unlike HIF-1α, which is considered the mediator of OXPHOS to glycolysis in hypoxic environments (Miska et al., 2019), limited evidence has shown the role of *EPAS1* in glycolysis and OXPHOS balance. Farsijani (Farsijani et al., 2016) demonstrated that HIF-2α increased the expression of glycolytic enzymes including hexokinase 2(Hk2), glucose transporter 1 (GLUT1), aldolase C (Aldoc), phosphoglycerate mutase 1 (Pgam1), 6-phosphofructo-2-kinase/fructose-2,6-biphosphatase 3 (PFKFB3), pyruvate kinase M2 (Pkm2), and lactate dehydrogenase A (Ldha), and promoted the synthesis of EPO in renal tubular epithelial cells during hypoxia in a VHL-dependent manner (Figure 3). Early elevated and later downregulated *EPAS1* are required for pluripotency induction of induced pluripotent stem cells (iPSC) by glycolysis reprogramming (Mathieu et al., 2014).

In addition to glycolysis, accumulated HIF-2α during chronic hypoxia impairs fatty acid β-oxidation in hepatocytes and ultimately leads to steatosis by suppressing the expression of carnitine palmitoyl transferase 1 (CPT-1) and proliferator-activated receptor-γ coactivator-1α (PGC-1α), rate-limiting enzymes for the β-oxidation pathway, which can be rescued after *EPAS1* knockdown (Liu et al., 2014). Similarly, Li (Li et al., 2021) revealed that HIF-2α transcriptally suppresses CPT-1 expression and prevents NLPR3 inflammasome activation, ultimately alleviating insulin resistance in chronic metabolic diseases. Qu (Qu et al., 2011) demonstrated that mice with liver-specific disruption of *Vhl* achieved *EPAS1* overexpression and presented steatosis accompanied by pro-inflammatory and fibrogenic cytokine overexpression. The latest finding by Mooli (Mooli et al., 2022) suggest that HIF-2α augments G-protein-coupled receptor 40 (GPR40) and 120 (GPR120) expressions in intestinal lipid-sensing L cells and induces GLP-1 secretion, regulating the lipid metabolism network. Moreover, constitutive activation of *EPAS1* impairs both fatty acid β-oxidation and lipogenesis by downregulating associated genes, whereas it promotes gene expression within the lipid storage and gluconeogenesis pathways (Rankin et al., 2009).

### 3.4 Inflammation

Microenvironmental hypoxia is commonly observed in various systemic inflammatory diseases, such as atherosclerosis, diabetes mellitus, inflammatory bowel diseases (IBD), cancers, and PH. In adipocytes (Figure 3), *EPAS1* overexpression upregulates pro-inflammatory cytokines, including interleukin (IL)-1β, monocyte chemotactic protein-1, and the nuclear factor κB (NF-κB) pathway (Lin et al., 2013). Mice with adipocyte-specific *EPAS1* overexpression developed lethal cardiac hypertrophy, which is considered the molecular mechanism linking obesity and cardiomyopathy. Xue (Xue et al., 2013; Kerber et al., 2020) reported that highly activated *EPAS1* in the intestinal epithelium of IBD patients and mice enhances tumor necrosis factor-α (TNF-α) expression and promotes experimental colitis by inducing TNF-α promoter activity through the myc-associated zinc-finger protein (MAZ) binding, sparing NF-κB pathway. Esophageal HIF-2α expression correlates with reflux esophagitis severity. Refluxed acidic bile salts stabilize epithelial HIF-2α promoting pro-inflammatory cytokine expression (Huo et al., 2017). In contrast, HIF-2α in renal endothelial cells suppresses inflammatory reactions and sustains recovery from ischemic kidney injury (Kapitsinou et al., 2014). The pro-inflammatory role of HIF-2α has also been reported in osteoarthritis (Inoue et al., 2015), hepatoma (Ahn et al., 2010), atopic dermatitis, and psoriasis (Tashiro et al., 2019).

## 4 Role of *EPAS1* in PH

### 4.1 Hypoxia signal and separate predominance between HIF-1 and HIF-2 in PH

**FIGURE 4:**
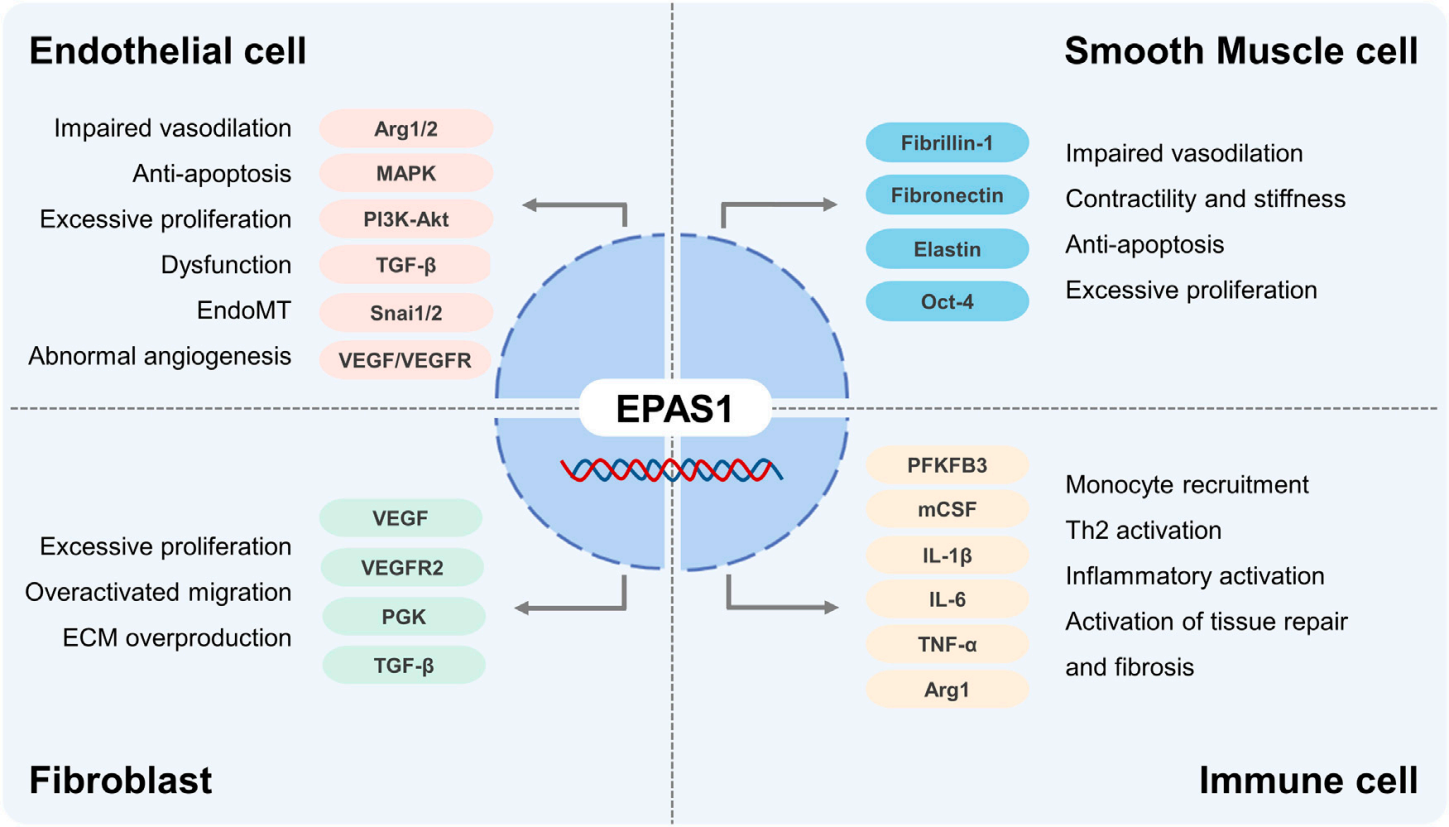
The role of EPAS1 in Pulmonary Hypertension.

Chronic hypoxia signaling plays a central role in the progression of pulmonary vascular remodeling, leading to irreversible PH and right heart failure. Investigators implied different cellular predominance of HIF-1 and HIF-2 in the development of PH. *EPAS1* exclusively governs the ECs dysfunction compared with *HIF-1α*, whereas in SMCs and fibroblasts, both *HIF-1α* and *EPAS1* orchestrate the pathogenesis of vascular remodeling (Waypa and Schumacker, 2019). Detailed aspects of *EPAS1* in PH pathogenesis are listed as below (Figure 4).

### 4.2 *EPAS1* in ECs

As the primary initiation of pulmonary vasculopathy, different molecular mechanisms focusing on EC dysfunction, including a spatiotemporal imbalance between apoptosis and proliferation, impairment of migration, sprouting and angiogenesis, methanological abnormalities, and endothelial to mesenchymal transition (EndoMT), explain the development of PH. HIF-2α was significantly elevated in the lung tissues, especially in ECs from patients with idiopathic PH, activates the cellular growth pathways, including MAPK, PI3K-Akt, VEGF, and transforming growth factor-β, increases pulmonary artery EC proliferation as well as collagen synthesis, and stimulates fibrotic remodeling of the pulmonary vasculature (Rimon et al., 2008; Fijalkowska et al., 2010; Bryant et al., 2016; Dai et al., 2018). Upregulated HIF-2α in ECs increases arginase 1 transcription and competes with the endothelial NO complex enzyme for L-Arginine, decreasing NO synthesis, the predominant vasodilator, promoting vascular remodeling and PH progression (Xu et al., 2004; Girgis et al., 2005; Cowburn et al., 2016). Research (Macias et al., 2021) also shown that Arg2 enzyme activity is elevated and correlated with *EPAS1* expression in vascular ECs derived from patients with PH. Using model of excessive accumulation of HIF-1 and HIF-2 proteins by inhibiting HIF prolyl hydroxylase domain-containing protein 2 (PHD2), investigators concluded that mice with EC knockout of *PHD2* developed severe pulmonary hypertension and right ventricular failure in a HIF-2 dependent manner, despite excessive accumulations of both HIF-1 and HIF-2 proteins (Kapitsinou et al., 2016). Dai (Dai et al., 2016) reported that mice with EC and bone marrow hematopoietic cells knockout of *PHD2* showed severe obliterative vascular remodeling and PH, by promoting SMCs proliferation through CXCL12 activation. HIF-2α (Tang et al., 2018) promotes ECs transition into mesenchymal myofibroblasts (EndoMT) by enhancing the expression of *Snai1/Snai2*, zinc-finger transcription factors family, and leads to remodeled pulmonary vasculopathy. EC-specific deletion of *EPAS1* may reduce the expression of ECM proteins, fibronectin, integrin receptors, and ET-B, disrupt cellular overgrowth, block plexiform formation, and ultimately inhibit hypoxia-induced pulmonary vascular remodeling (Hu et al., 2019). Rodents exposed in chronic hypoxia plus inhibition of VEGFR signal by Sugen5416 present as severe pulmonary obliterative vasculopathy and ventricular failure, whereas selectively disrupting VEGFR2 Y949, the tyrosine at residual 949 of VEGFR2, prevents vascular permeability with subsequent myeloid cells infiltration and pulmonary arterioles muscularization (Zhou et al., 2022). HIF-2α regulates angiogenesis in ECs by promoting transcription of VEGF-A/C and VEGFR1/2 pathway, regardless VEGF-B and VEGFR3 regulated by HIF-1α(Downes et al., 2018). Although the correlation between HIF-2α and VEGF/VEGFR signal pathway in PH development is rarely reported, but the paradoxical influence in ECs and pulmonary vascular function is still required clarification.

### 4.3 *EPAS1* in SMCs

The hyperplastic proliferation of pulmonary arterial SMCs (PASMCs) is attributed to the core pathogenesis of pulmonary vascular remodeling. Different studies showed that HIF-1α is overactivated in PASMCs in PH, whereas *EPAS1* is rarely reported (Dai et al., 2018). In the study of Xin Yi Chan (Chan et al., 2021), gain-of-function mutation of *EPAS1* in SMCs increases the expression of fibrillin-1, fibronectin, and elastic fiber components elastin (*ELN*) *via* activating endothelin 1 (*EDN1*) transcription, which contributes to the contractility and stiffness of SMCs and development of pulmonary hypertension in mice. Another data support from Raghavan (Raghavan et al., 2012) pointed out that HIF-2α stimulated PASMCs proliferation by upregulating the expression of transcription factor Oct-4 (Firth et al., 2010). Conclusively, the effects of *EPAS1* on PASMCs in PH are still undetermined and correlations among different vascular cells governed by HIF families pend further investigations.

### 4.4 *EPAS1* in fibroblasts

Adventitial fibroblast activation and transition to myofibroblasts, the major origin of collagen and ECM synthesis, are believed to promote vasculopathy in PH. With inhibition of HIF-1α and *EPAS1* using RNA interference technology, Eul (Eul et al., 2006) reported that the proliferative response of adventitial fibroblasts relies only on HIF-2α by activation of VEGF, VEGFR2, Phosphoglycerate kinase (PGK) and TGFβ, while the migratory response is correlated with both HIF-1α and HIF-2α. Moreover, mutation of the *VHL* gene at codon 200 results in cellular HIF-2α accumulation, further induces pulmonary fibroblasts activation with ECM overproduction, and, thereby promoting pulmonary fibrosis (Hickey et al., 2010).

### 4.5 *EPAS1* in immune cells

Myeloid cells, especially the monocyte-macrophage lineage, are increasingly reported to contribute to PH pathogenesis (Lim et al., 2013; He et al., 2020). During the early stage of PH, circulatory monocytes are recruited to the pulmonary interstitial space, infiltrating as a pro-inflammatory phenotype and mediating EC dysfunction and PASMC proliferation (Tian et al., 2013; Florentin et al., 2018). In addition to HIF-1α, Wang (Wang et al., 2021) demonstrated that myeloid activation of *PFKFB3* impels inflammatory macrophage differentiation *via* the HIF-2α pathway. HIF-2α upregulates macrophage colony-stimulating factor (M-CSF) expression and promotes monocyte perivascular infiltration and differentiation into inflammatory macrophages (Wang et al., 2018), secreting cytokines including IL-1β, IL-6, IL-8, IL-13, IL-18, and TNF-α, which are believed to be related to PH severity and prognosis (Groth et al., 2014). Interestingly, phenotypic alterations in anti-inflammatory/profibrotic macrophages participate in the late stage of pulmonary vascular remodeling. Few studies have revealed the role of *EPAS1* in anti-inflammatory macrophages in PH. Li and colleagues (Li et al., 2021) argued that NLRP3 inflammasome activation of M1 could be suppressed by HIF-2α*,* thereby preventing insulin resistance. Takeda (Takeda et al., 2010) explained that *EPAS1* induced by T helper 2 cytokines (IL-4) specifically promoted arginase 1 expression during M2 macrophage polarization. Despite 48% structural similarity, several data revealed the opposing regulatory roles of HIF-1α and HIF-2α on macrophages phenotype dominance which destine outcomes of inflammatory diseases and malignant tumor (Takeda et al., 2010; Eubank et al., 2011).

## 5 Strategies targeting on *EPAS1*

Surveys by Scheuermann (Scheuermann et al., 2009; Scheuermann et al., 2013). have identified a hydrophobic cavity at PAS-B domain of EPAS1 which can be occupied by artificial ligands, disrupting the EPAS1-ARNT heterodimer formation, inhibiting the transcription of target genes, and potentially reverse the development of PH. Zimmer (Zimmer et al., 2008) reported a small molecule inhibitor of HIF-2α, C76, facilitates the binding of Iron Regulatory Protein 1 (IRP1) to the IRE of *EPAS1* message and abolishes *EPAS1* translation, showing as a potential strategy ameliorating the vascular remodeling and right ventricular hypertrophy in PH. Coincidentally, Hu demonstrated (Hu et al., 2019) mice with *EPAS1* inducible deletion by antisense oligonucleotides (EPAS1-ASO) exhibited a decreased right ventricular hypertrophy index, reduced vascular remodeling and increased survival of PH. PT2567, an orally bioavailable compound of HIF-2α inhibitor, significantly diminished the early monocytes recruitment, pulmonary vascular cells proliferation, right ventricular remodeling, and plasma nitrite concentration in rats during hypoxia induced PH development. *In vitro* study, PT2567 reduces arginase1 activity induced by HIF-2α and attenuates inflammation and dysfunction of ECs(Macias et al., 2021). These data indicate the potential role of pharmacological agents targeting on HIF-2α in the interventional strategies of PH.

## 6 Conclusion and perspectives

The current standard management of PH mainly focuses on regenerating normal vasomotor function instead of preventing vascular remodeling, which causes the high mortality and low quality of life. A new interventional strategy reversing pulmonary vascular remodeling is imperative for the treatment of PH. As a key regulator of PH, HIF-2α is involved in pulmonary vascular remodeling, erythropoiesis, the inflammatory response, and hypoxic metabolism during PH. Therapies targeting or blocking HIF-2α pathways have proven advantages in opposing vascular remodeling progression both *in vivo* and *in vitro* (Zheng et al., 2022). However, most exploration lines within the preclinical stage, and credible and valid data from clinical trials are required for further verification. In addition, more investigations on the biological function of the *EPAS1* regulation network are required to develop a comprehensive picture of the pathophysiology of PH.
